# Neo-X-Linked Chromosome Polymorphism: Cytogenetic Insights from *Passalites nemorivagus* (Mammalia, Cervidae) †

**DOI:** 10.3390/ani15172557

**Published:** 2025-08-30

**Authors:** Raquel Muhlbeier Bonato, Agda Maria Bernegossi, Eluzai Dinai Pinto Sandoval, Halina Cernohorska, Miluse Vozdova, José Maurício Barbanti Duarte

**Affiliations:** 1 Deer Research and Conservation Center (NUPECCE), School of Agricultural and Veterinarian Sciences, São Paulo State University (UNESP), Jaboticabal 14884-900, Brazil; raquel.bonato@unesp.br (R.M.B.); agda.bernegossi@unesp.br (A.M.B.);; 2 Central European Institute of Technology, Veterinary Research Institute, 621 00 Brno, Czech Republicmiluse.vozdova@vri.cz (M.V.)

**Keywords:** cytogenetics, Cervidae, chromosome rearrangement, sex chromosome, conservation

## Abstract

Chromosomal instability plays a significant role in karyotype evolution and speciation in mammalian groups with notable intraspecific chromosomal variation. The Cervidae family, known for its rapid karyotypic evolution due to chromosomal fragility, shows substantial chromosomal diversity, making it a focal point for studies on chromosomal evolution, particularly with respect to conservation and taxonomic classification. The Amazon gray brocket deer (*Passalites nemorivagus*) exhibits pronounced chromosomal polymorphism, including two distinct sex chromosome systems: the ancestral XX/XY system and a new system due to an X–autosome fusion (neo-X), where males present XY1Y2. This variation is intriguing, especially given that the effects on hybrids have not been previously reported. This study uses bovine whole-chromosome painting (WCP) and BAC probes to document karyotypic variation in *P. nemorivagus*. A male with the XY system and a heterozygous autosomal Robertsonian fusion was paired with a female with neo-X chromosomes, and the resulting female offspring displayed an X–autosome fusion in heterozygosity. The females in this study, hybrids for the sex system, exhibited estrus, copulated, and both gave birth to offspring. This characterization is the first step in investigating the effects of sex chromosome system variation on hybrid viability and fertility, and provides insights into the reproductive biology of Neotropical deer.

## Simple Summary

Chromosomal changes play a key role in the evolution and diversity of species. Within the deer family, known for rapid and varied chromosome variations, the Amazon gray brocket deer presents two distinct sex chromosome systems, a standard and a complex system due to a rearrangement involving an X chromosome. This study investigated these chromosome variations by analyzing a male and female deer with different sex chromosome systems and their female offspring. The hybrid females displayed normal reproductive behavior and successfully produced offspring. These results provide new insights into how differences in sex chromosomes affect reproduction and viability in mammals. Understanding these genetic mechanisms is important for the conservation and proper classification of these Neotropical deer.

## 1. Introduction

Structural chromosomal variations can lead to significant phenotypic effects and are widely recognized for their potential role in evolutionary divergence. The concept of chromosomal speciation proposes that hybrid dysfunction may result from the formation of unbalanced gametes or the suppression of recombination, with the accumulation of genetic incompatibilities over time ultimately leading to reproductive isolation [1,2]. In this context, chromosomal instability contributes to karyotype evolution by generating barriers to gene flow and facilitating lineage differentiation [2,3]. Comparative cytogenetics has therefore become a powerful tool for exploring how karyotypic changes influence mammalian speciation [3].

While intraspecific chromosomal rearrangements in mammals are found to be rather random in mass data surveys of agricultural and medical cytogenetics [4], different types of chromosomal variability are typical for many natural species and supraspecific groups [5]. The Cervidae family, known for its high karyotypic evolutionary differentiation rate due to pronounced chromosomal fragility [6], shows considerable chromosomal diversity, with diploid numbers (2n) ranging from 6 in female *Muntiacus muntjak vaginalis* to 70 in many deer species such as *Subulo gouazoubira* [7,8,9]. This diversity has evolved through tandem and Robertsonian translocations of acrocentric chromosomes, including those involving sex chromosomes [10]. Such variation has made Cervidae a focal point for studies on chromosomal evolution, particularly because of its significance for conservation and taxonomic classification [9].

The Amazonian brown brocket deer *(Passalites nemorivagus),* the smallest gray brocket deer occurring in the Amazon region, is in particular genetically interesting [11]. It has displayed chromosomal polymorphisms involving Robertsonian and tandem fusions, resulting in variations in diploid number (2n) from 66 to 70 and fundamental number of arms (FN) from 69 to 72 [11,12,13]. Another notable genetic aspect is phylogenetic analysis based on mitochondrial DNA fragments, which revealed a well-structured differentiation of the species into three distinct clades, with strong geographical association, restricted to specific regions of endemism within the Amazon [11,12]. The pronounced karyotypic variation, in association with the phylogenetic divergence, suggests the presence of a cryptic species complex in *P. nemorivagus*, implying reproductive isolation among certain populations [13]. Moreover, the most remarkable cytogenetic feature is the presence of two distinct sex chromosome systems within the species [11]. Classical cytogenetic studies have suggested to result from an X–autosome tandem fusion, with some individuals exhibiting the simple and ancestral sex chromosome system (XX/XY), and others possessing a multiple sex chromosome system due to a neo-X with an autosome fusion (XX/XY_1_Y_2_) [11,13,14]. In this context, the topotype of the species, whose karyotype was mapped using bovine-derived artificial bacterial chromosome (BAC) probes selected based on the mapped karyotype of *S. gouazoubira* [9], exhibited a simple sex chromosome system [11].

Despite generally being considered evolutionarily conserved in eutherian mammals, the X chromosome is also susceptible to structural modifications, which may be underrepresented in cytogenetic studies [15]. The emergence of multiple sex chromosome systems is generally attributed to Robertsonian fusions between autosomes and sex chromosomes, and the cases that occur due to tandem fusions are little discussed [16,17]. Hughes et al. [15] observed that 152 of 6640 species of mammals present variant sex chromosomes, and within the order Artiodactyla, only the families Bovidae and Cervidae have species with these variations. Specifically in Cervidae, the XY_1_Y_2_ system due to a neo-X has been documented in the genera *Muntiacus* [18], *Elaphodus* [19], *Mazama* [20,21,22], and *Passalites*, the latter being the only from the Blastocerina subtribe to exhibit this system [11,13,14].

Although X–autosome fusions could have the potential to cause significant deleterious effects, the relatively frequent occurrence in mammals suggests that these rearrangements are compatible with meiotic processes [23]. Several studies have reported differences in the meiotic behavior of gonosomal regions involved in evolutionarily fixed sex–autosome fusions and translocations in mammals [24,25,26]. However, in ruminants, even when sex chromosomes are fused with autosomes, meiotic silencing is restricted to the gonosomal portion and does not affect the fused autosomes. Furthermore, no evidence suggests that sex–autosome fusions lead to somatic dysfunctions or reproductive limitations in these animals [27].

Regardless of the existing genetic knowledge about the Amazonian gray brocket deer, there remains a significant gap in general information about the species, mainly due to the difficulty of accessing their natural habitat and the small number of individuals kept in captivity [11,28]. In Brazil, the only confirmed captive population is housed at the Deer Research and Conservation Center (NUPECCE), thus, the ex situ populations serve as a safeguard for wild species and provides an essential source of biological research data that is otherwise inaccessible under challenging and unpredictable field conditions [29].

Despite the common variation of the standard XY system in mammals, the observation of two different systems in *P. nemorivagus* is intriguing, and the effects on hybrids have never been reported. The objective of this study is to report a previously undocumented karyotypic variation in *P. nemorivagus*, observed in the offspring from breeding studies conducted at NUPECCE. In this study, one male with the simple sex chromosome system (XY) and a heterozygous autosomal Robertsonian translocation was paired with a female that possessed a multiple sex chromosome system (neo-X) and no Robertsonian fusion. The karyotypic characterization of the resulting offspring is the first step to investigate the effects of sex chromosome system variation on hybrid viability and fertility. In addition, it will provide valuable insights into the Neotropical deer reproductive biology and may contribute to broader knowledge about chromosomal instability and its role in evolution of Neotropical deer species.

## 2. Materials and Methods

Captive Breeding. This study was approved by the Ethics and Animal Welfare Committee of the School of Agricultural and Veterinary Studies, Jaboticabal, SP, Brazil (protocol 005433/19, valid from June 2019 to May 2024). All individuals were housed and carefully managed under human care at the Deer Research and Conservation Center (NUPECCE), ensuring a high standard of welfare. A male *P. nemorivagus*, identified as CATFAP 04, was born in captivity at NUPECCE and was mated multiple times with a female, identified as T370, originating from Pará, Brazil. Mating occurred following the detection of estrus, characterized by mucus discharge and the female’s receptivity to the male. Pregnancy was confirmed through the absence of estrus 21 days after mating [30]. These pairings led to the birth of four offspring: two males (T391 and T398) and two females (T425 and T435). The offspring were kept with their mother until weaning, ensuring a natural adaptation to solid feeding.

Skin collection. We collected skin tissue samples from each individual (parents and offspring when adults). Animals were chemically restrained with an association of xylazine hydrochloride 2% (Rompum 1 mg/kg i.m.) and ketamine 10% (Dopalen 7 mg/mgi.m.), and the skin fragment was obtained from the inner region of the pelvic limbs [31]. The area was washed with 1% triclosan antiseptic soap (Soapex 1%), trichotomized, and cleaned three times with 70% ethanol. The suture was performed after collection and the individuals were observed until anesthesia recovery. The skin fragments of 4 cm^2^ were transported immediately to the laboratory.

Cytogenetic analysis. Skin fragments were then transferred to flasks containing culture medium (Dulbecco’s modified Eagle medium [DMEM] enriched with 20% fetal bovine serum [FBS]), and incubated at 37 °C with 5% CO_2_. After achieving fibroblast cell confluence, the material underwent colchicine treatment, followed by a hypotonic solution, and was fixed in Carnoy’s solution [32]. Parental and offspring karyotypes were determined by subjecting the chromosomal preparations to conventional Giemsa staining.

Whole-chromosome painting (WCP) probes. Bovine chromosomes were microdissected by PALM Microlaser system (Carl Zeiss MicroImaging GmbH, Munich, Germany), and the chromosomal DNA was then amplified by DOP-PCR (degenerate oligonucleotide primed polymerase chain reaction) [33]. Probe labeling was performed during the secondary PCR with Green-dUTP or Orange-dUTP (Abbott, North Chicago, IL, USA) [34]. The WCP probes were then used for identification of chromosomes involved in the X–autosome fusion in the two female offspring.

BAC probes. We also used bovine-derived artificial bacterial chromosome (BAC) probes into the karyotype of the individuals, to understand better the direction of the fusions. BAC clones were selected from the CHORI-240 library based on the NCBI ARS-UCB1.2 Assembly data, and obtained from BACPAC Genomics, Emeryville, CA, USA. DNA were extracted using an adapted protocol from the method included in Wizard^®^ Plus SV Minipreps DNA Purification Systems [9]. Probes were selected considering the mapped *P. nemorivagus* karyotype, to test for the fusions already described occurring in the species [11]. We used the following BAC probes: centromeric region of BTA 19 (BAC 130E10), marked with Green-dUTP (Abbott, North Chicago, IL, USA), and p arm of BTA X (BAC 159O16), marked with digoxigenin-11-dUTP (Roche, Mannheim, Germany).

Fluorescence in situ hybridization. Fluorescence in situ hybridization procedures were carried out as described in Vozdova et al. [35]. Probes labeled with biotin-16-dUTP were detected using streptavidin-Cy5 (Invitrogen/Molecular Probes, Camarillo, CA, USA), whereas those labeled with digoxigenin-11-dUTP were detected using anti-digoxigenin-rhodamine (Roche Diagnostics, Indianapolis, IN, USA). Chromosomes were counterstained with 4′,6-diamidino-2-phenylindole (DAPI). Hybridization signals were examined using Zeiss Axio Imager Z2 fluorescence microscope with appropriate fluorescent filters (Carl Zeiss Microimaging GmbH, Jena, Germany); images were captured by a CoolCube CCD camera (MetaSystems, Altlussheim, Germany) and analyzed by ISIS (MetaSystems, Altlussheim, Germany).

## 3. Results

**Figure 1:**
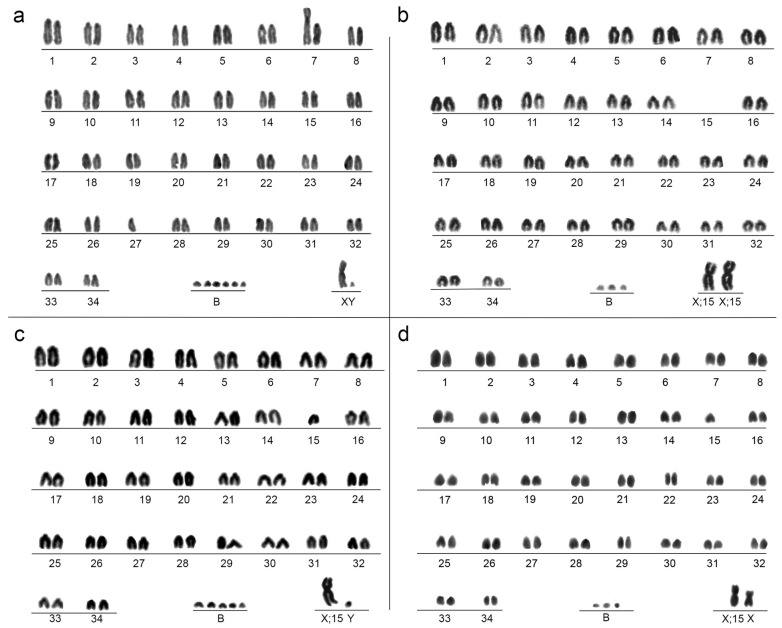
Karyotypes after conventional Giemsa staining of the studied animals. (**a**) Father CATFAP 04, (**b**) mother T370, (**c**) male offspring (T398), representing also the male sibling T391, and (**d**) female offspring (T425), representing also the female sibling T435.

**Figure 2:**
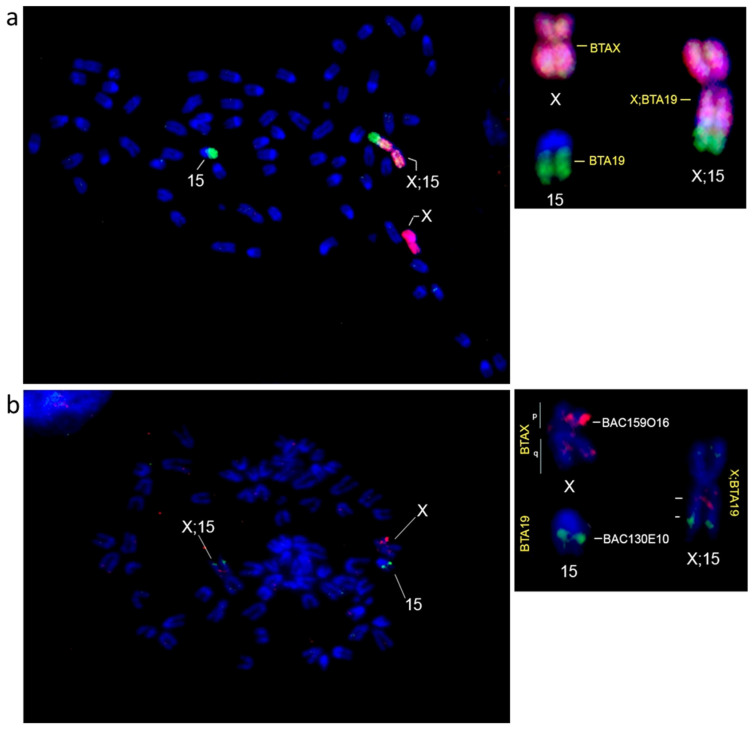
FISH results on chromosomes of one of the female offspring studied (T425), showing the heterozygous sex chromosome system, where one X chromosome is normal (ancestral) and the other presents the tandem fusion (X;15, i.e., neo-X). (**a**) WCP FISH result using BTAX (red) and BTA19 (green) probes, marking, respectively, the chromosomes X and 15. (**b**) BAC FISH result using BAC 130E10 (chromosome 15, green) and BAC 159O16 (p arm of chromosome X, red).

Giemsa staining revealed that the father’s (CATFAP 04) karyotype (2n = 69; FN = 71) exhibited mostly acrocentric autosomes and one large submetacentric resulting from a Robertsonian fusion. The X and Y chromosomes were both submetacentric (Figure 1a). The karyotype of the mother (T370) (2n = 68; FN = 70) did not exhibit the Robertsonian fusion, but possessed the neo-X system, resulting from an X–autosome fusion, inferred by the absence of a pair of autosomes (Figure 1b). None of the four offspring (two males—2n = 69; FN = 70, and two females—2n = 69; FN = 71) inherited the Robertsonian fusion from their father (Figure 1c,d). All individuals displayed multiple B chromosomes, varying from three to six between cells of the same individual and between individuals. While Giemsa staining revealed clear chromosomal morphologies, it proved less effective in distinguishing between the normal X chromosome and the fused X chromosome. In contrast, FISH provided more precise information. In the female offspring, one X chromosome hybridized only with the X-specific probes, while the other showed a fusion between the centromeric end of chromosome 15, which is homologous to BTA19, linearly connected to the distal terminal p arm of X chromosome. This highlights a centromere–telomere tandem fusion between chromosome 15 and the ancestral X, originating the neo-X in *P. nemorivagus* (Figure 2).

The analysis of the sex chromosomes showed that both male offspring inherited the X;15 from their mother, and the female offspring inherited the X;15 from their mother and the normal X from their father, resulting in the heterozygous sex chromosome system, never reported before in Artiodactyla (Figure 2). Due to the X;15 fusion, one of the X chromosomes in the female offspring is significantly larger than its counterpart.

## 4. Discussion

Cytogenetic studies are essential to understanding the evolutionary processes and reproductive dynamics in Neotropical deer [21]. Among the various chromosomal features observed in these species, B chromosomes represent an intriguing element of karyotypic diversity that has been reported across multiple genera [9,11]. These chromosomes are considered supernumerary, exhibiting non-Mendelian inheritance patterns and typically not resulting in visible phenotypic effects [36]. Previous studies report intraindividual variability ranging from zero to six B chromosomes in the genus *Mazama*, zero to seven Bs in *P. nemorivagus*, and zero to three Bs in *S. gouazoubira* [9,11,37]. The variation observed in the individuals of our study is consistent with these patterns supporting the high degree of B chromosome variability within Neotropical deer.

The X chromosome of *P. nemorivagus* has a submetacentric morphology, which differs from the ancestral karyotype retained in *Subulo gouazoubira* by a pericentric inversion and a change in the centromeric position [9,11]. In brocket deer, the chromosomal instability is frequent due to fragile regions prone to nonrandom breakage and potential sites of rearrangements [6]. Our results obtained through FISH support the findings of Morales-Donoso et al. [11], which identified chromosomes 7 and 27 as being involved in the Robertsonian fusion. However, whereas their study reported all specimens exhibiting a simple sex chromosome system (XX/XY), we found one centromere–telomere tandem fusion between chromosome 15 and the ancestral X, which originated the neo-X chromosome in *P. nemorivagus* specimens presenting a multiple sex chromosome system. In addition, the Pseudoautosomal region is located on the terminal region of the q arm in this species, indicating that the fusion occurred in a region unrelated to X–Y recombination [11].

The females presented in our study are individuals with balanced chromosomal rearrangements, which involve no gain or loss of genetic material, and consequently, they are phenotypically normal [38]. The X–autosome fusion has also been observed in other Neotropical deer species. Molecular cytogenetic studies with BAC probes have shown that, aside from the differences observed with *S. gouazoubira*, as previously mentioned, there is a noticeable conservation of orthology regarding the X chromosome across several species studied thus far. These species include the red deer complex (*Mazama americana* and *M. rufa*) [39], *Odocoileus pandora* [40], *Bisbalus citus* [41], *Mazama temama* [42], and *P. nemorivagus* [13]. The observed differences are primarily related to species that have neo-X chromosomes, which resulted from the fusion of the ancestral X chromosome with autosomes. In the future, studies with more refined scales, such as genomic studies, could better confirm this conservation.

Sex chromosome–autosome translocations and fusions are relatively common in mammals, despite the potential for deleterious effects [23], and this phenomenon is one of the mechanisms by which neo-sex chromosomes evolve [17]. Such translocations involving both the X and Y chromosomes have been documented across various species, including rodents, bats, primates, marsupials, bovids, cervids, and humans [24,26,43,44,45,46,47]. While cattle and humans present clinical conditions associated with these translocations [46,48], other species seem to have developed adaptations that allow them to maintain fertility [26,27]. In these cases, such alterations are not only tolerated but can even become fixed through mechanisms such as sexual antagonism, heterozygote advantage, and genetic drift in neo-sex chromosomes [17,49,50]. Regarding the X chromosome, the order Artiodactyla stands out as one of the notable cases where this chromosome underwent significant intrachromosomal and interchromosomal changes, including inversions, centromere shift, heterochromatic variation, and X–autosomal translocations [48,51,52]. Moreover, the X chromosome in Capreolinae, the Cervidae tribe to which *P. nemorivagus* is allocated, is less conserved and more prone to rearrangements than in Cervinae [10].

In this study, we observed that male offspring inherited the X–autosomal fusion (neo-X) from their mother, and the homologous autosome along with the Y chromosome from their father, setting up the multiple sex chromosome system, as seen in free-living specimens. In contrast, only females exhibited a distinct karyotype with the tandem fusion in heterozygosity. The existence of two or more configurations of sex chromosomes within one species is rare in eutherians, and in Artiodactyla this phenomenon has been mentioned only in Soemmerring’s gazelle, *Nanger soemmerringii* (Bovidae), in the Tufted deer *Elaphodus cephalophus,* and the Amazon gray brocket deer *P. nemorivagus* (both Cervidae), briefly discussing it through classical cytogenetics [13,19,53,54]. However, despite the well-established presence of multiple sex chromosome systems in these species, this is the first report of a heterozygous sex chromosome system in the order, as a result of the mating of individuals with different karyotypes. In this context, the lack of investigations in the meiotic fates of different sex chromosome morphologies is still an open question about their role in speciation [15].

Studies have demonstrated that the presence of a multiple sex chromosome system in males does not interfere with normal meiosis and spermatogenesis in animals [24,26,55,56]. Studying the synaptonemal complex, Vozdova et al. [27] investigated six species of the family Bovidae with sex chromosome translocations. Their findings demonstrated that meiotic silencing, associated with the inactivation of sex chromosomes, occurs normally in all species studied. A similar study with phyllostomid bats also demonstrates that multiple sex chromosomes can successfully bypass the risk of meiotic arrest and infertility [26]. Other studies demonstrate that the fused autosome is isolated from the spreading of gonosomal chromosome inactivation [56,57].

Females with two copies of such fusion chromosomes also undergo complete homologous synapsis during meiotic prophase, ensuring proper meiotic progression [58]. However, in a female heterozygous for an X–autosome fusion, with one normal X chromosome, anomalous pairing during meiosis may occur. This can lead to gametogenesis failures and the production of unbalanced gametes, as previously observed in hybrid cytotypes of *M. americana* [14]. These hybrids, which were heterozygous for one tandem fusion between autosomes, exhibited the highest rate of unbalanced spermatozoa among all *Mazama* males analyzed [14]. The fertilization of unbalanced gametes may lead to trisomies or monosomies, conditions generally incompatible with life [38].

The theory that *P. nemorivagus* is a cryptic species complex is based on phylogenetic studies and its cytogenetic polymorphism, which could explain the different sex systems and imply reproductive isolation among certain populations, due to meiosis problems previously discussed [13,37]. However, during routine management at NUPECCE, the females in this study, hybrids for the sex system, exhibited estrus, copulated, and both gave birth to offspring. This outcome highlights the absence of a prezygotic barrier between the individuals, but we still cannot exclude completely the possibility of postzygotic isolation and a certain degree of decline in fertility in these females.

A well-clarified cryptic species complex has been identified within the genus *Mazama*, the red brocket deer (*M. americana* and *M. rufa*), characterized by significant karyotypic divergence due to tandem fusions, centric fusions, inversions, and X–autosomal fusions, with diploid numbers ranging from 42 to 53 [22,37,39,59]. In this case, the genetic differences were supported by reproductive alterations, with studies evaluating the impact of chromosomal polymorphisms as reproductive barriers, and showing the existence of well-established reproductive isolation among the most distant karyotype lineages [14,60,61]. Thus, these results evidenced that the accumulation of heterozygous tandem fusion in autosomes is detrimental to reproduction, and suggested an efficient postzygotic barrier. However, a single tandem fusion did not prevent reproduction between individuals, although it did cause a decrease in sperm quality [14,60,61].

**Figure 3:**
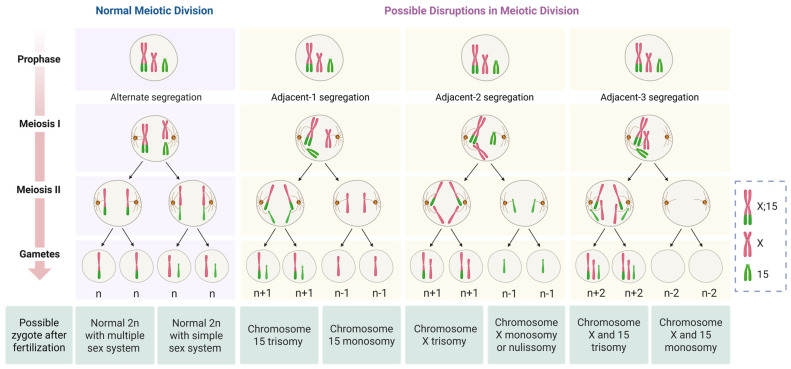
Segregation patterns during meiosis and representation of possible oocytes produced from a carrier with one fused X;15 (neo-X) and one normal X chromosome, and the zygote formed after fertilization with a normal male (XY or XY1Y2), which can be normal, trisomic, or monosomic for one or both of the chromosomes involved in the translocation.

It is possible to infer that a tandem fusion between autosomes may cause more severe disruptions in meiotic division than a fusion between a sex chromosome and an autosome. This could be explained by the inactivation of one X chromosome as part of gene dosage compensation in somatic cells, as described by Lyon [62], probably justifying a better tolerance of animal cells to sex chromosome abnormalities [48]. Therefore, by virtue of this mechanism, even if the females related in this study produce unbalanced gametes with an extra or lack of X chromosome, and they were fertilized, the offspring could manage a possible aneuploidy more effectively. A fraction of unbalanced gametes would be tolerated, but they would result in offspring with Klinefelter (XXY_1_Y_2_, XXY), Turner syndromes (XO), or trisomy of X (XXX), which are infertile or sterile animals, thereby excluding them from conservation efforts, either in situ or ex situ (Figure 3).

Therefore, even after producing offspring, it would be ideal to evaluate quantitatively the reproductive potential of the daughters. However, female gametes are difficult to obtain for research purposes because of the need for invasive procedures such as follicle puncture or surgery to collect oocytes or ovarian cortex tissue [60,63,64]. Furthermore, these techniques are challenging to perform on wild animals, as they require adaptation from methods used on better-studied species [60]. In human, several studies using FISH have described sperm cells with X chromosomal aberration [63]. In contrast, comparable analyses in female ovarian cells with X chromosomal aberrations remain scarce, as does detailed information on the reproductive implications of aneuploidy in these cells [63,64].

## 5. Conclusions

The present study provides the first documented case of an X–autosome fusion in heterozygosity in *P. nemorivagus*, which has not been previously observed in either free-living or captive individuals of this species or any other artiodactyl. This unique finding contributes valuable insights into variant sex chromosome systems in mammals, particularly regarding the evolutionary dynamics of the X chromosome. Furthermore, this chromosomal rearrangement challenges prior assumptions and provides new perspectives on how chromosomal instability may drive karyotypic diversification and potentially influence reproductive isolation, thereby shaping species’ evolutionary trajectories. These findings establish a foundation for future research exploring the impact of such genetic traits on biodiversity in mammals. This article is a revised and expanded version of a paper entitled “Genética da conservação: polimorfismo sexual no Veado-roxo e implicações para o manejo reprodutivo em cativeiro”, which was presented at 48º Congresso da Associação de Zoológicos e Aquários do Brasil (AZAB), Brazil, in June 2025 [65].

## Data Availability

The datasets generated during and/or analyzed during the current study are available from the corresponding author on reasonable request.

## References

[1] Rieseberg L.H. (2001). Chromosomal Rearrangements and Speciation. Trends Ecol. Evol..

[2] Faria R., Navarro A. (2010). Chromosomal Speciation Revisited: Rearranging Theory with Pieces of Evidence. Trends Ecol. Evol..

[3] Graphodatsky A.S., Trifonov V.A., Stanyon R. (2011). The Genome Diversity and Karyotype Evolution of Mammals. Mol. Cytogenet..

[4] Ducos A., Berland H.-M., Bonnet N., Calgaro A., Billoux S., Mary N., Garnier-Bonnet A., Darré R., Pinton A. (2007). Chromosomal Control of Pig Populations in France: 2002–2006 Survey. Genet. Sel. Evol..

[5] Dobigny G., Britton-Davidian J., Robinson T.J. (2017). Chromosomal Polymorphism in Mammals: An Evolutionary Perspective. Biol. Rev. Camb. Philos. Soc..

[6] Vargas-Munar D.S.F., Sarria-Perea J.A., Duarte J.M.B. (2010). Different Responses to Doxorubicin-Induced Chromosome Aberrations in Brazilian Deer Species. Genet. Mol. Res..

[7] Wurster D.H., Benirschke K. (1970). Indian Muntjac, *Muntiacus muntjak*: A Deer with a Low Diploid Chromosome Number. Science.

[8] Fontana F., Rubini M. (1990). Chromosomal Evolution in Cervidae. Biosystems.

[9] Bernegossi A.M., Vozdova M., Cernohorska H., Kubickova S., Galindo D.J., Kadlcikova D., Rubes J., Duarte J.M.B. (2022). Cytogenetic Mapping of Cattle BAC Probes for the Hypothetical Ancestral Karyotype of the Family Cervidae. Cytogenet. Genome Res..

[10] Proskuryakova A.A., Ivanova E.S., Makunin A.I., Larkin D.M., Ferguson-Smith M.A., Yang F., Uphyrkina O.V., Perelman P.L., Graphodatsky A.S. (2023). Comparative Studies of X Chromosomes in Cervidae Family. Sci. Rep..

[11] Morales-Donoso J.A., Vacari G.Q., Bernegossi A.M., Sandoval E.D.P., Peres P.H.F., Galindo D.J., De Thoisy B., Vozdova M., Kubickova S., Barbanti Duarte J.M. (2023). Revalidation of *Passalites* Gloger, 1841 for the Amazon Brown Brocket Deer *P. nemorivagus* (Cuvier, 1817) (Mammalia, Artiodactyla, Cervidae). ZooKeys.

[12] Duarte J.M.B., González S., Maldonado J.E. (2008). The Surprising Evolutionary History of South American Deer. Mol. Phylogenet. Evol..

[13] Fiorillo B.F., Sarria-Perea J.A., Abril V., Duarte J.M.B. (2013). Cytogenetic Description of the Amazonian Brown Brocket *Mazama nemorivaga* (Artiodactyla, Cervidae). Comp. Cytogenet..

[14] Galindo D.J., Martins G.S., Vozdova M., Cernohorska H., Kubickova S., Bernegossi A.M., Kadlcikova D., Rubes J., Duarte J.M. (2021). Chromosomal Polymorphism and Speciation: The Case of the Genus *Mazama* (Cetartiodactyla; Cervidae). Genes.

[15] Hughes J.J., Lagunas-Robles G., Campbell P. (2024). The Role of Conflict in the Formation and Maintenance of Variant Sex Chromosome Systems in Mammals. J. Hered..

[16] Kitano J., Peichel C.L. (2012). Turnover of Sex Chromosomes and Speciation in Fishes. Environ. Biol. Fishes.

[17] Yoshida K., Kitano J. (2012). The contribution of female meiotic drive to the evolution of neo-sex chromosomes. Evolution.

[18] Pathak S., Lin C.C. (1981). Synaptonemal Complex of the Sex-Autosome Trivalent in a Male Indian Muntjac. Chromosoma.

[19] Cao X., Jiang H., Zhang X. (2005). Polymorphic Karyotypes and Sex Chromosomes in the Tufted Deer *(Elaphodus cephalophus)*: Cytogenetic Studies and Analyses of Sex Chromosome-Linked Genes. Cytogenet. Genome Res..

[20] Bernegossi A.M., Cartes J.L., Cernohorska H., Kubickova S., Vozdova M., Caparroz R., González S., Duarte J.M.B. (2023). Resurrection of the Genus *Subulo* Smith, 1827 for the Gray Brocket Deer, with Designation of a Neotype. J. Mammal..

[21] Aquino C.I., Abril V.V., Duarte J.M.B. (2013). Meiotic Pairing of B Chromosomes, Multiple Sexual System, and Robertsonian Fusion in the Red Brocket Deer *Mazama americana* (Mammalia, Cervidae). Genet. Mol. Res..

[22] Peres P.H.F., Luduvério D.J., Bernegossi A.M., Galindo D.J., Nascimento G.B., Oliveira M.L., Sandoval E.D.P., Vozdova M., Kubickova S., Cernohorska H. (2021). Revalidation of *Mazama rufa* (Illiger 1815) (Artiodactyla: Cervidae) as a Distinct Species out of the Complex *Mazama americana* (Erxleben 1777). Front. Genet..

[23] Dobigny G., Ozouf-Costaz C., Bonillo C., Volobouev V. (2004). Viability of X-Autosome Translocations in Mammals: An Epigenomic Hypothesis from a Rodent Case-Study. Chromosoma.

[24] Deuve J.L., Bennett N.C., Ruiz-Herrera A., Waters P.D., Britton-Davidian J., Robinson T.J. (2008). Dissection of a Y-Autosome Translocation in *Cryptomys hottentotus* (Rodentia, Bathyergidae) and Implications for the Evolution of a Meiotic Sex Chromosome Chain. Chromosoma.

[25] Borodin P.M., Fedyk S., Chętnicki W., Torgasheva A.A., Pavlova S.V., Searle J.B., Searle J.B., Polly P.D., Zima J. (2019). Meiosis and Fertility Associated with Chromosomal Heterozygosity. Shrews, Chromosomes and Speciation.

[26] Rahn M.I., Noronha R.C., Nagamachi C.Y., Pieczarka J.C., Solari A.J., Sciurano R.B. (2016). Protein Markers of Synaptic Behavior and Chromatin Remodeling of the Neo-XY Body in Phyllostomid Bats. Chromosoma.

[27] Vozdova M., Ruiz-Herrera A., Fernandez J., Cernohorska H., Frohlich J., Sebestova H., Kubickova S., Rubes J. (2016). Meiotic Behaviour of Evolutionary Sex-Autosome Translocations in Bovidae. Chromosome Res..

[28] De Oliveira M.L., De Faria Peres P.H., Gatti A., Morales-Donoso J.A., Mangini P.R., Duarte J.M.B. (2020). Faecal DNA and Camera Traps Detect an Evolutionarily Significant Unit of the Amazonian Brocket Deer in the Brazilian Atlantic Forest. Eur. J. Wildl. Res..

[29] Comizzoli P., Holt W.V. (2019). Breakthroughs and New Horizons in Reproductive Biology of Rare and Endangered Animal Species. Biol. Reprod..

[30] Oliveira M.E.F., Zanetti E.D.S., Cursino M.S., De Fátima Carvalho Peroni E., Rola L.D., Feliciano M.A.R., Canola J.C., Duarte J.M.B. (2016). First Live Offspring of Amazonian Brown Brocket Deer *(Mazama nemorivaga)* Born by Artificial Insemination. Eur. J. Wildl. Res..

[31] Oliveira C.C.C., Queiroz Vacari G., Maurício Barbanti Duarte J. (2024). A Method to Freeze Skin Samples for Cryobanks: A Test of Some Cryoprotectants for an Endangered Deer. Biopreserv. Biobank..

[32] Verma R., Babu A. (1995). Human Chromosomes: Principles & Techniques.

[33] Telenius H., Carter N.P., Bebb C.E., Nordenskjöld M., Ponder B.A., Tunnacliffe A. (1992). Degenerate Oligonucleotide-Primed PCR: General Amplification of Target DNA by a Single Degenerate Primer. Genomics.

[34] Kubickova S., Cernohorska H., Musilova P., Rubes J. (2002). The Use of Laser Microdissection for the Preparation of Chromosome-Specific Painting Probes in Farm Animals. Chromosome Res..

[35] Vozdova M., Kubickova S., Cernohorska H., Fröhlich J., Vodicka R., Rubes J. (2019). Comparative Study of the Bush Dog *(Speothos venaticus)* Karyotype and Analysis of Satellite DNA Sequences and Their Chromosome Distribution in Six Species of Canidae. Cytogenet. Genome Res..

[36] Camacho J.P.M., Sharbel T.F., Beukeboom L.W. (2000). B-Chromosome Evolution. Phil. Trans. R. Soc. Lond. B.

[37] Abril V.V., Carnelossi E.A.G., González S., Duarte J.M.B. (2010). Elucidating the Evolution of the Red Brocket Deer *Mazama americana* Complex (Artiodactyla; Cervidae). Cytogenet. Genome Res..

[38] Huang N., Zhou J., Lu W., Luo L., Yuan H., Pan L., Ding S., Yang B., Liu Y. (2023). Characteristics and Clinical Evaluation of X Chromosome Translocations. Mol. Cytogenet..

[39] Bernegossi A.M., Galindo D.J., Peres P.H.F., Vozdova M., Cernohorska H., Kubickova S., Kadlcikova D., Rubes J., Duarte J.M.B. (2024). Comparative Karyotype Analysis of the Red Brocket Deer (*M. americana* Sensu Lato and *M. rufa)* Complex: Evidence of Drastic Chromosomal Evolution and Implications on Speciation Process. J. Appl. Genet..

[40] Sandoval E.D.P., Bernegossi A.M., Gallina S., Reyna-Hurtado R., Duarte J.M.B. (2023). Cytogenetic, Molecular, and Morphological Characterization of *Odocoileus pandora* (Merriam, 1901) (Artiodactyla, Cervidae). Can. J. Zool..

[41] Sandoval E.D.P., Jędrzejewski W., Molinari J., Vozdova M., Cernohorska H., Kubickova S., Bernegossi A.M., Caparroz R., Duarte J.M.B. (2024). Description of *Bisbalus*, a New Genus for the Gray Brocket, *Mazama cita* Osgood, 1912 (Mammalia, Cervidae), as a Step to Solve the Neotropical Deer Puzzle. Taxonomy.

[42] Sandoval E.D.P., Bernegossi A.M., Gallina S., Reyna-Hurtado R., Duarte J.M. (2024). Molecular Cytogenetics Markers Reveal the Existence of a Cryptic Complex of *Mazama temama* Species. Therya.

[43] Hayman D. (1989). Marsupial Cytogenetics. Aust. J. Zool..

[44] Toder R., O’Neill R.J.W., Wienberg J., O’Brien P.C.M., Voullaire L., Marshall-Graves J.A. (1997). Comparative Chromosome Painting between Two Marsupials: Origins of an XX/XY1Y2 Sex Chromosome System. Mamm. Genome.

[45] Steinberg E.R., Bressa M.J., Mudry M.D. (2022). Sex Chromosome Systems in Neotropical Primates: What Have We Learnt so Far from Cytogenetics and Genomics?. J. Evol. Biol..

[46] Di-Battista A., Favilla B.P., Zamariolli M., Nunes N., Defelicibus A., Armelin-Correa L., da Silva I.T., Reymond A., Moyses-Oliveira M., Melaragno M.I. (2023). Premature Ovarian Insufficiency Is Associated with Global Alterations in the Regulatory Landscape and Gene Expression in Balanced X-Autosome Translocations. Epigenet. Chromatin.

[47] Povill C., de Oliveira M.B., de Abreu F.V.S., de Oliveira R.L., Perini F.A., Monticelli C., Bueno C., dos Santos E., Pissinatti A., Bonvicino C.R. (2023). Genetic Diversity and Insights into the Distribution of Brown Howler Monkeys (*Alouatta guariba* Group) (Atelidae, Alouattinae). Int. J. Primatol..

[48] Iannuzzi A., Parma P., Iannuzzi L. (2021). Chromosome Abnormalities and Fertility in Domestic Bovids: A Review. Animals.

[49] Charlesworth B., Wall J.D. (1999). Inbreeding, Heterozygote Advantage and the Evolution of Neo-X and Neo-Y Sex Chromosomes. Proc. R. Soc. B Biol. Sci..

[50] Rice W.R. (1984). Sex Chromosomes and the Evolution of Sexual Dimorphism. Evolution.

[51] Robinson T.J., Harrison W.R., Ponce de León F.A., Davis S.K., Elder F.F. (1998). A Molecular Cytogenetic Analysis of X Chromosome Repatterning in the Bovidae: Transpositions, Inversions, and Phylogenetic Inference. Cytogenet. Cell Genet..

[52] Proskuryakova A.A., Kulemzina A.I., Perelman P.L., Makunin A.I., Larkin D.M., Farré M., Kukekova A.V., Lynn Johnson J., Lemskaya N.A., Beklemisheva V.R. (2017). X Chromosome Evolution in Cetartiodactyla. Genes.

[53] Shi L., Yang F., Kumamoto A. (1991). The Chromosomes of Tufted Deer (*Elaphodus cephalophus*). Cytogenet. Cell Genet..

[54] Steiner C.C., Charter S.J., Goddard N., Davis H., Brandt M., Houck M.L., Ryder O.A. (2015). Chromosomal Variation and Perinatal Mortality in San Diego Zoo Soemmerring’s Gazelles. Zoo. Biol..

[55] Solari A.J., Pigozzi M.I. (1994). Fine Structure of the XY Body in the XY1Y2 Trivalent of the Bat *Artibeus lituratus*. Chromosome Res..

[56] Noronha R.C.R., Nagamachi C.Y., O’Brien P.C.M., Ferguson-Smith M.A., Pieczarka J.C. (2010). Meiotic Analysis of XX/XY and Neo-XX/XY Sex Chromosomes in Phyllostomidae by Cross-Species Chromosome Painting Revealing a Common Chromosome 15-XY Rearrangement in Stenodermatinae. Chromosome Res..

[57] Noronha R.C.R., Nagamachi C.Y., Pieczarka J.C., Marques-Aguiar S., Assis M.F.L., Barros R.M.D.S. (2004). Meiotic Analyses of the Sex Chromosomes in Carolliinae-Phyllostomidae (Chiroptera): NOR Separates the XY1Y2 into Two Independent Parts. Caryologia.

[58] Ashley T. (2002). X-Autosome Translocations, Meiotic Synapsis, Chromosome Evolution and Speciation. Cytogenet. Genome Res..

[59] Cifuentes-Rincón A., Morales-Donoso J.A., Sandoval E.D.P., Tomazella I.M., Mantellatto A.M.B., De Thoisy B., Duarte J.M.B. (2020). Designation of a Neotype for *Mazama americana* (Artiodactyla, Cervidae) Reveals a Cryptic New Complex of Brocket Deer Species. ZooKeys.

[60] Cursino M.S., Salviano M.B., Abril V.V., Zanetti E.d.S., Duarte J.M.B. (2014). The Role of Chromosome Variation in the Speciation of the Red Brocket Deer Complex: The Study of Reproductive Isolation in Females. BMC Evol. Biol..

[61] Salviano M.B., Cursino M.S., Zanetti E.D.S., Abril V.V., Duarte J.M.B. (2017). Intraspecific Chromosome Polymorphisms Can Lead to Reproductive Isolation and Speciation: An Example in Red Brocket Deer (*Mazama americana*). Biol. Reprod..

[62] Lyon M.F. (1961). Gene Action in the X-Chromosome of the Mouse (*Mus. musculus* L.). Nature.

[63] Chatziparasidou A., Christoforidis N., Samolada G., Nijs M. (2015). Sperm Aneuploidy in Infertile Male Patients: A Systematic Review of the Literature. Andrologia.

[64] Nadesapillai S., van der Velden J., Braat D., Fleischer K., Peek R. (2023). Exploring X Chromosomal Aberrations in Ovarian Cells by Using Fluorescence In Situ Hybridization. J. Vis. Exp..

[65] Bonato R.M., Bernegossi A.M., Sandoval E.D.P., Cernohorska H., Vozdova M., Duarte J.M.B. Genética da conservação: Polimorfismo sexual no Veado-roxo e implicações para o manejo reprodutivo em cativeiro. Proceedings of the Anais do 48º Congresso da Associação de Zoológicos e Aquários do Brasil (AZAB).

